# Updating salamander datasets with phenotypic and stomach content information for two mainland *Speleomantes*

**DOI:** 10.1038/s41597-021-00931-w

**Published:** 2021-06-09

**Authors:** Enrico Lunghi, Fabio Cianferoni, Simone Giachello, Yahui Zhao, Raoul Manenti, Claudia Corti, Gentile Francesco Ficetola

**Affiliations:** 1 Key Laboratory of the Zoological Systematics and Evolution, Institute of Zoology, Chinese Academy of Sciences, Beichen West Road 1, 100101 Beijing, China; 2 Museo di Storia Naturale, Università degli Studi di Firenze, Via Romana 17, 50125 Firenze, Italy; 3 Natural Oasis, via di Galceti 141, 59100 Prato, Italy; 4 Unione Speleologica Calenzano, Piazza della Stazione 1, 50041Calenzano, Firenze, Italy; 5 Istituto di Ricerca sugli Ecosistemi Terrestri (IRET), Consiglio Nazionale delle Ricerche (CNR), Via Madonna del Piano, 50019 Sesto Fiorentino, Firenze, Italy; 6 Dipartimento di Scienze e Politiche Ambientali, Università degli Studi di Milano, via Celoria 26, 20133 Milano, Italy; 7 Laboratorio di Biologia Sotterranea “Enrico Pezzoli”, Parco Regionale del Monte Barro, 23851 Galbiate, Italy; 8 Université Grenoble Alpes, CNRS, Laboratoire d’Écologie Alpine (LECA), CS 40700, 38058 Grenoble, France

**Keywords:** Herpetology, Population dynamics

## Abstract

European plethodontid salamanders (genus *Speleomantes*; formerly *Hydromantes*) are a group of eight strictly protected amphibian species which are sensitive to human-induced environmental changes. Long-term monitoring is highly recommended to evaluate their status and to assess potential threats. Here we used two low-impact methodologies to build up a large dataset on two mainland *Speleomantes* species (*S. strinatii* and *S. ambrosii*), which represents an update to two previously published datasets, but also includes several new populations. Specifically, we provide a set of 851 high quality images and a table gathering stomach contents recognized from 560 salamanders. This dataset offers the opportunity to analyse phenotypic traits and stomach contents of eight populations belonging to two *Speleomantes* species. Furthermore, the data collection performed over different periods allows to expand the potential analyses through a wide temporal scale, allowing long-term studies.

## Abstract (summary)

**Table:** 

Measurement(s)	Gastric Content • Image
Technology Type(s)	light microscopy • Digital Photography
Sample Characteristic - Organism	Speleomantes • Hydromantes
Sample Characteristic - Environment	Subterranean • karst cave • mine
Sample Characteristic - Location	Italy • Region of Liguria • Municipality of Genova • Province of La Spezia

Machine-accessible metadata file describing the reported data: 10.6084/m9.figshare.14346176

## Background & Summary

European cave salamanders are a group of eight amphibians endemic to Italy and to a small part of the French Provence^1^, all belonging to the genus *Speleomantes*^1^ (formerly considered *Hydromantes*). Three species (*S. strinatii*, *S. ambrosii* and *S. italicus*) are distributed along the Northern and Central Apennines (only *S. strinatii* naturally extends its range also in France), while the other five (*S. flavus*, *S. supramontis*, *S. imperialis*, *S. sarrabusensis* and *S. genei*) are endemic to Sardinia^1^. European cave salamanders are fully terrestrial and lack lungs (Lanza *et al*., 2006). These features force *Speleomantes* to select only specific microclimatic conditions: they need high moisture and relatively cold temperatures to survive^2^. Therefore, *Speleomantes* often inhabit subterranean environments^3,4^, where their preferred microclimatic conditions are realized^5^. Nonetheless, subterranean environments also may be chosen by *Speleomantes* because predator pressure is lower if compared to epigean ones^6,7^. Indeed, *Speleomantes* likely represent one of the apex predators in these environments^8^, preying on a wide array of taxa^9^. *Speleomantes*’ narrow eco-physiological requirements, combined with their limited distributions and high site fidelity^1,10^, make these species very sensitive to human-induced effects and susceptible to extinction^11,12^; all *Speleomantes* species are therefore strictly protected^13,14^.

Improving our knowledge of species at risk of extinction is fundamental to assess their potential threats and to guarantee their survival^15,16^. For example, prolonged monitoring may help to understand the impact of specific environmental changes^17–19^, allowing to forecast future scenarios and promptly act to protect endangered species^20–22^. In this regard, the production of comparable datasets through time is of key importance^9,23,24^. However, data collection may not always be an easy task. Species can occur in habitats that pose challenges to human exploration, as for subterranean environments, where sampling and progression require considerable effort and specific technical skills^25–27^. Subterranean habitats are not only hard to find or explore, but their peculiar environmental conditions (e.g., cold temperatures, high moisture, narrow space) might challenge surveyor’s stamina with a negative impact on the data recorded^25,28^. Another limit to data collection can be determined by the techniques used to collect information. For example, the old-fashioned research methods involving the sacrifice/harm of individuals are now widely condemned and avoided, especially when concerning protected species^29,30^. Therefore, there is an increasing trend in the use of new harmless alternative approaches^31,32^.

We here describe a new database reporting data on two endangered *Speleomantes* species that can be handled only under specific national authorizations (see Acknowledgments). This dataset includes information on the population structure, phenotypic traits and diet of individuals belonging to two mainland *Speleomantes* species: *S. strinatii* and *S. ambrosii*. The information gathered here was collected adopting methodologies that limit negative impact on individuals, and can be combined with the two previously published datasets on these species^9,23^, extending the available information in space and time. In this work we gathered data from new populations to cover more area of the species’ range, but we also repeated the surveys in previously visited populations, thus providing temporal series of information allowing long-term studies focusing on populations but also on single individuals as well^33,34^. Specifically, we here describe a dataset composed of two types of data: images and stomach contents. High quality images allow extrapolation of data on multiple phenotypic traits (e.g., size, morphology, coloration), obtaining information on the overall population traits and on single individuals as well^23,33,35^. In the era of digitization^36,37^, this new “living” digital catalogue (i.e., collection composed by photos of living organisms) will partially replace the natural history museum collections, becoming, at least for some animal groups, an alternative that not only spare animals lives, but also overcome classic limits such as space needed to store specimens and readiness to be used by the worldwide scientific community with no costs^37^. Another advantage of “living” collections is its repeatability, namely the possibility that individuals can be digitally collected multiple times, allowing to perform long term studies on single individuals and populations as well. The data related to stomach contents can be analysed to study the species’ trophic niche and the multiple related traits^34,38,39^. Nonetheless, comparing datasets produced over different time allows to assess potential variation affecting specific species traits and infer on the possible causes^40,41^.

## Methods

### Surveyed sites

**Fig. 1:**
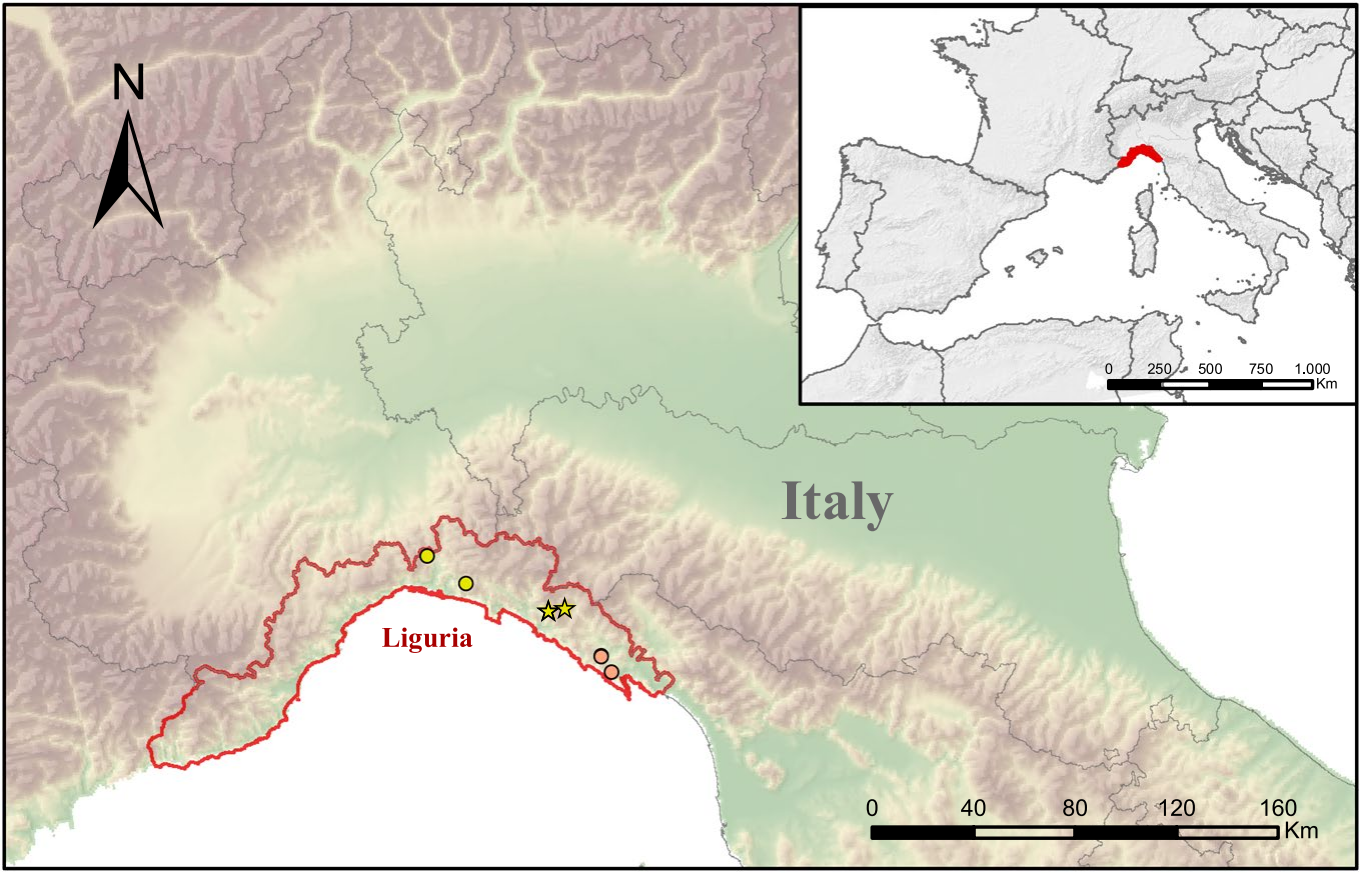
Map of the surveyed populations. Yellow labels indicate the surveyed sites for *Speleomantes strinatii*, while red ones those surveyed for *S. ambrosii*. Stars indicate the artificial subterranean environments, while circles identify natural ones. Precise coordinates are not shown to increase populations protection^58^.

We surveyed eight subterranean sites, three artificial mines and five natural caves (Fig. 1); all these sites fall within the species’ natural range^1^. Surveys were performed in 2020, between 9 am and 6 pm during warm and sunny days, periods in which subterranean abundance of *Speleomantes* is the highest^42^. All sites were surveyed in July, while for six of them surveys were also repeated in September (Table 1). We performed extensive research of *Speleomantes* within the subterranean sites^43^, covering areas where the exploration was possible without speleological equipment. Salamanders were captured and placed in drilled plastic boxes waiting to be checked. When salamanders sampling was finished, we proceeded with the data collection following this order: (*i*) assessment of the presence/absence of the mental gland for the identification of adult males (see Fig. 1a in^35^); (*ii*) record of body weight using a digital scale (precision 0.01 g); (*iii*) stomach flushing (see below); (*iv*) photo shooting (see below). Salamanders were then released in their collection points.

**Table 1:** Updating photos and stomach contents datasets for two mainland *Speleomantes* salamanders: data from summer 2020.

Column	Data description	Typology of data
1	ID	The salamander’s database code
2	Site	“Cave” or “Mine”
3–4	Latitude and Longitude	Low resolution coordinates of the site
5	Population	The population code
6–7	Region and Province	The relevant information for each site
8–9	Month and Year	The period in which the salamander was captured
10	Species	The species to which the individual belongs
11	N_photo	The unique file number corresponding to each individual
12	Weight	Indicates the salamander’s weight (g)
13	Mental_gland	Indicates the presence (1) or absence (0) of the male mental gland
14	Eggs	Indicates whether eggs where visible through the salamander’s belly (yes/no)
15	Scale_bar	Indicates the size of the picture scale bar (mm)
16	Condition	Indicates if stomach was empty (1) or not (0)
17	Not_identifiable	Indicates if stomach content was identifiable (0) or not (1)
18 to 34	Prey typology	For each prey typology the total number of recognized items is reported

Detailed information related to the photographed salamanders and to their stomach contents^45^.

### Stomach flushing

Stomach flushing is a technique enabling to inspect amphibians stomach contents without harming individuals^29,44^. A detailed description of this methodology is provided in Lunghi, *et al*.^9^. Preserved stomach contents were examined in the lab using an optical microscope, and undigested prey items (or part of them; see^9^) were recognized at the order level; Staphylinidae (Coleoptera) and Formicidae (Hymenoptera) insect families were considered separately. When possible, also arthropods’ different stages where considered as independent prey category. Stomach contents were considered: “empty”, if no prey item was observed; “not-identifiable”, if the advanced stage of digestion prevented the identification to the order level; “full”, if at least one prey item was recognizable. Recognized prey were counted following the method described in Lunghi, *et al*.^9^. Prey items were rarely integer and the identification was often based on fragments. This condition hampered any potential standardized measurement of prey volume using representative geometric polygons. Stomach flushing was performed on a subsample of the captured salamanders (Table 1).

### Photo shooting

In a dark area of the subterranean environment, captured salamanders were dorsally photographed inside a white soft-box to obtain an homogeneous illumination of the subject and reduce shadows^23^. Before each photo session, a photo was shot to a Pantone colour card X-Rite Colorcheker Passport 2 placed into the soft-box to correctly calibrate the colours and light of photos during post-production^23^. We then photographed salamanders next to a plastic ruler to have a standardize size reference. Please refer to^23^ for additional information on this method.

## Data Records

The dataset (Photos and stomach contents of two mainland Italian *Speleomantes* salamanders: data from summer 2020^45^) can be downloaded from *figshare* and consists of:

1. 854 photos of salamanders from two mainland *Speleomantes* species: *S. strinatii* (S_strinatii4, 28 in July; S_strinatii1, 47 in July and 73 in September; S_strinatii2, 15 in July and 10 in September; S_strinatii5, 118 in July and 88 in September, S_strinatii6, 26 in July) and *S. ambrosii* (S_ambrosii2, 108 in July and 98 in September; S_ambrosii3, 21 in July and 51 in September; S_ambrosii4, 59 in July and 112 in September). The code adopted to label populations (name of the species + number indicating the population) enables to combine the present dataset with two previously published (see Usage Notes).
2. 565 salamanders stomach contents, subdivided in 313 individuals with empty stomach, 37 with non-recognizable contents, and 215 individuals with full stomach.
3. 978 recognized prey items belonging to 17 different prey categories (Pulmonata, Araneae, Pseudoscorpiones, Opiliones, Julida, Isopoda, Entomobryomorpha, Orthoptera, Blattodea, Hymenoptera, Formicidae, Coleoptera, Diptera, Diptera_larva, Archaeognatha, Speleomantes_skin, Haplotaxida).
4. NA means no specific data existing.

Detailed explanation of dataset Photos and stomach contents of two mainland Italian *Speleomantes* salamanders: data from summer 2020^45^ is given in Table 1.

## Technical Validation

This dataset provides data on two strictly protected amphibian species^13^. Salamanders were sampled following protocols aiming to avoid the spread of potential pathogens^46^; specifically, we used disposable gloves and disinfected with bleach equipment and boots before changing location. During each month, all surveys were performed within 4 days to limit the variation of environmental conditions which may alter the local ecological opportunity^38,41^. To limit pseudoreplication, each site was surveyed only once per month. On the other hand, the two surveys performed on the same populations during different months (July and September) create the condition to test additional hypotheses, like the assessment of temporal variability on salamanders’ trophic niche^41^, or even to employ specific software to individually recognize salamanders^47,48^ and focus future research on single individuals as well. A blinded stomach contents analysis was performed to limit possible bias^49^. The methodology used to shoot photos enables the production of standardised high quality images with low impact on the species^23,50^. The white calibration before each session avoided potential divergence in light condition and thus, providing standardised pictures. This method allows the creation of a “living” digital collection of *Speleomantes*^23^, a method that does not only avoid the sacrifice of animals (and thus the related stochastic effects conditioning the evolution of populations) but it is also repeatable. For future updates we will try to include an estimation of prey volume, preferably using methodologies allowing to directly measure the volume (i.e., immerging residuals in a liquid).

## Usage Notes

The first part of this dataset is composed of high quality images of individuals of *Speleomantes strinatii* and *S. ambrosii* from dorsal view. We suggest the use of the program ImageJ to extrapolate salamanders morphometrics and to estimate their snout-vent length^35^, a fundamental parameter to distinguish juveniles from adults^1^. The images can be also used in R environment (http://www.R-project.org/) to perform analysis on multiple phenotypic traits^51,52^. Considering that the dorsal pattern of *Speleomantes* does not change through the time^33^, it can be used as natural mark to individually recognize salamanders^48,53^. The repeated surveys performed on the same locations were thought to test the efficacy of specific software to automatically recognize *Speleomantes* salamanders^47,53^. It has been observed that the ventral pattern of the mainland *S. strinatii* can be used to individually recognize salamanders, and that software can be employed to do it automatically^54^. However, only the three mainland species have visible pigments on their ventral side^1^, thus alternative methods are needed for analyses embracing all *Speleomantes* (i.e. including the five Sardinian species). Furthermore, the recognition of *Speleomantes* using their dorsal pattern limits individuals’ handling, a potential source of both stress and pathogens^50,55–57^. The population S_strinatii2 in the previously published dataset^23^ included two nearby sites, the S_strinatii2 and S_strinatii5 shown here. To combine these data, the actual S_strinatii2 contains the previous 20 photos (1045074–1045113) and the actual S_strinatii5 the other 20 (1045114–1045142). Furthermore, also the population S_strinatii3 in the previous dataset^23^ includes 2 different nearby sites; we therefore suggest to split it in S_strinatii3 with the first 33 photos (1045032–1045056) and in S_strinatii7 with the other 10 (1045057–1045069).

The second part of the dataset is provided in CSV format and is ready to be analysed with R. Populations are codified following^23^. To combine the data on stomach contents with the previous dataset:^9^ S_ambrosii2 = Cave_ambrosii1, S_ambrosii3 = Cave_ambrosii3, S_ambrosii4 = Cave_ambrosii2. The data on stomach contents allows to assess different characteristics of the populations trophic niche^34,38,41^. However, only after verifying whether the single individuals were captured during the two different surveys, it is possible to evaluate the variation of individuals’ trophic niche over time.

## Data Availability

No code was used in this study.
